# Rotarex Debulking in a Subtotal Occlusion of the Common Femoral Artery After Intimal Protrusion Caused by a Vascular Closure (StarClose) Device

**DOI:** 10.1002/ccr3.70689

**Published:** 2025-08-14

**Authors:** Theodoros Moysidis, Daniel Herzenstiel, Grigorios Korosoglou

**Affiliations:** ^1^ Vascular Medicine Knappschaftskrankenhaus Dortmund Dortmund Germany; ^2^ Cardiology and Vascular Medicine GRN Hospital Eberbach Eberbach Germany; ^3^ Cardiology and Vascular Medicine GRN Hospital Weinheim Weinheim Germany

**Keywords:** acute limb ischemia, drug‐coated balloon, peripheral artery disease, rotational thrombectomy

## Abstract

We present a case of a 72‐year‐old female patient who presented with resting pain in her right limb 3 weeks after coronary intervention via femoral access. Duplex sonography and angiography revealed subtotal occlusion of the right common femoral artery, due to vessel wall protrusion into the lumen, caused by the vascular closure (StarClose) device, which was used for puncture site closure during the initial coronary intervention. The patient was successfully treated with 8F Rotarex debulking and subsequent drug‐coated balloon angioplasty without stent placement. Duplex sonography after 4 weeks showed biphasic flow in the right limb down to the distal pedal arteries.

**Summary**

- Vascular closure device complications are rare but may occur during coronary or peripheral interventions.
- Interventionalists may consider endovascular revascularization as an option for the management of such complications in selected cases and based on shared decision making with the patients.

## 1 Introduction

Endovascular techniques emerged as the first option for treatment of peripheral artery disease (PAD) in most patients with lifestyle‐limiting claudication and chronic limb‐threatening ischemia [1]. Due to these developments and the application of endovascular revascularization in complex PAD [2, 3, 4], more complications may occur during treatment [5].

Thus, the appropriate endovascular management of these complications may represent an important element for the further establishment and acceptance of such procedures. Herein, we present a case of an iatrogenic subtotal occlusion of the right common femoral artery (CFA) after percutaneous coronary intervention (PCI), which was managed minimally invasively by debulking and balloon angioplasty and without the need for stent placement or open repair.

## 2 Case History/Examination

A 72‐year‐old female patient was referred to our department due to stable angina during exertion, Canadian Cardiovascular Society class III, for several months. ECG and troponin testing were negative, and echocardiography exhibited normal left‐ventricular function without regional wall motion abnormalities. Written informed consent was obtained from the patient. The patient had no history of coronary artery disease, PAD, or cerebrovascular disease. She had arterial hypertension and hyperlipidemia. Stress echocardiography exhibited inducible inferior wall motion abnormality. Therefore, the patient was scheduled for elective coronary angiography, and PCI of the right coronary artery was performed due to a high‐grade (> 90%‐diameter) stenosis. PCI was performed using a 6F sheath without complications, and a StarClose clip device (Abbott Vascular, Redwood, City, CA, USA) was used for closure of the puncture site in the right groin at the end of the procedure. The further course of the patient was uneventful, and she could be discharged the next day. However, 3 weeks after the PCI, the patient was re‐admitted due to resting pain in her right limb.

### 2.1 Differential Diagnosis, Investigations, and Treatment

**FIGURE 1:**
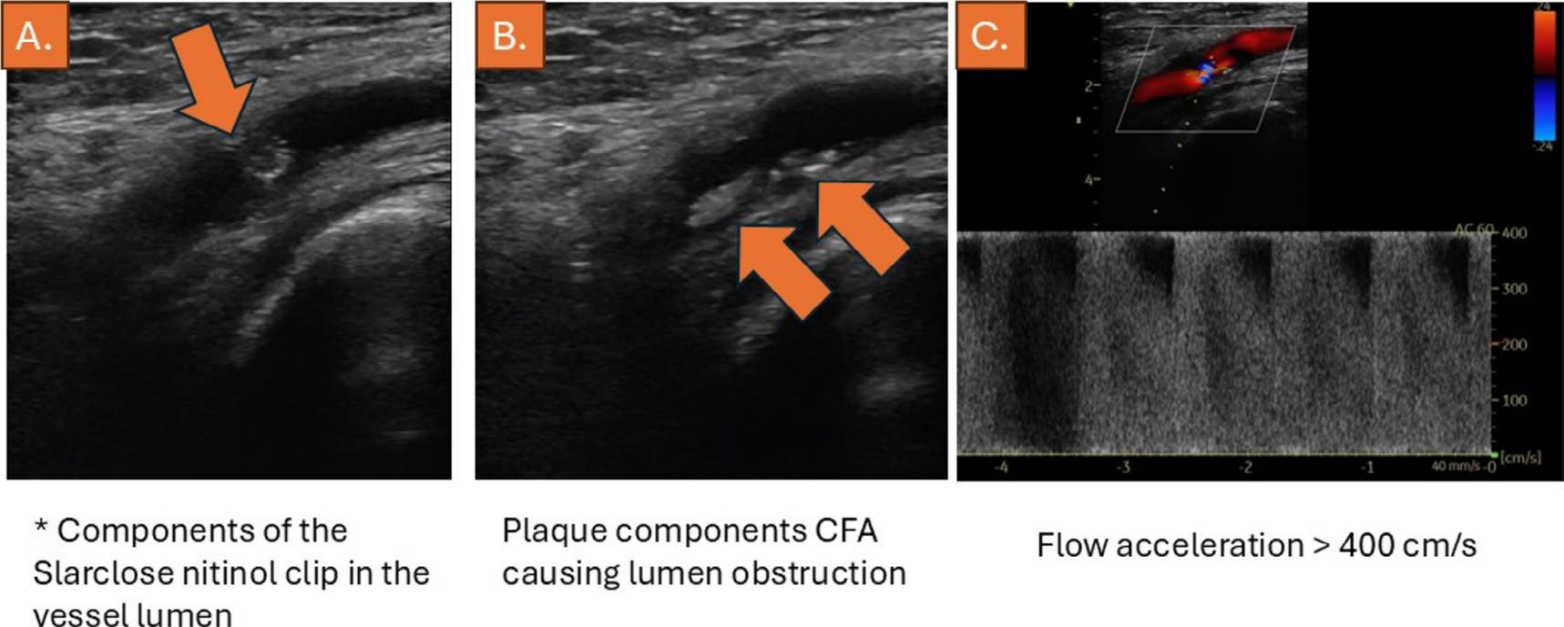
Duplex sonography, showing the StarClose nickel–titanium clip within the vessel lumen (A), which together with plaque components (B), results in subtotal occlusion of the artery with flow acceleration of over 400 cm/s (C).

On clinical examination, a faint pulse was registered in the right groin, whereas no pulses were palpable in her right foot. Normal pulses were present in the contralateral left limb. Duplex sonography showed parts of the StarClose nickel‐titanium clip within the vessel lumen, which together with plaque components and suspected organized thrombus led to a strong flow acceleration of over 400 cm/s, corresponding to high grade > 90% stenosis (Figure 1A–C). Subsequently, diminished monophasic flow patterns were noted in the superficial femoral artery (SFA). Due to conclusive clinical symptoms and duplex sonography findings, an iatrogenic occlusion of the CFA was suspected. Since the duplex images were considered diagnostic, computed tomography angiography was not deemed necessary prior to invasive angiography and treatment. An embolic occlusion of the artery was also considered, but was deemed less probable since the patients had a sinus rhythm by ECG and no history of atrial fibrillation.

**FIGURE 2:**
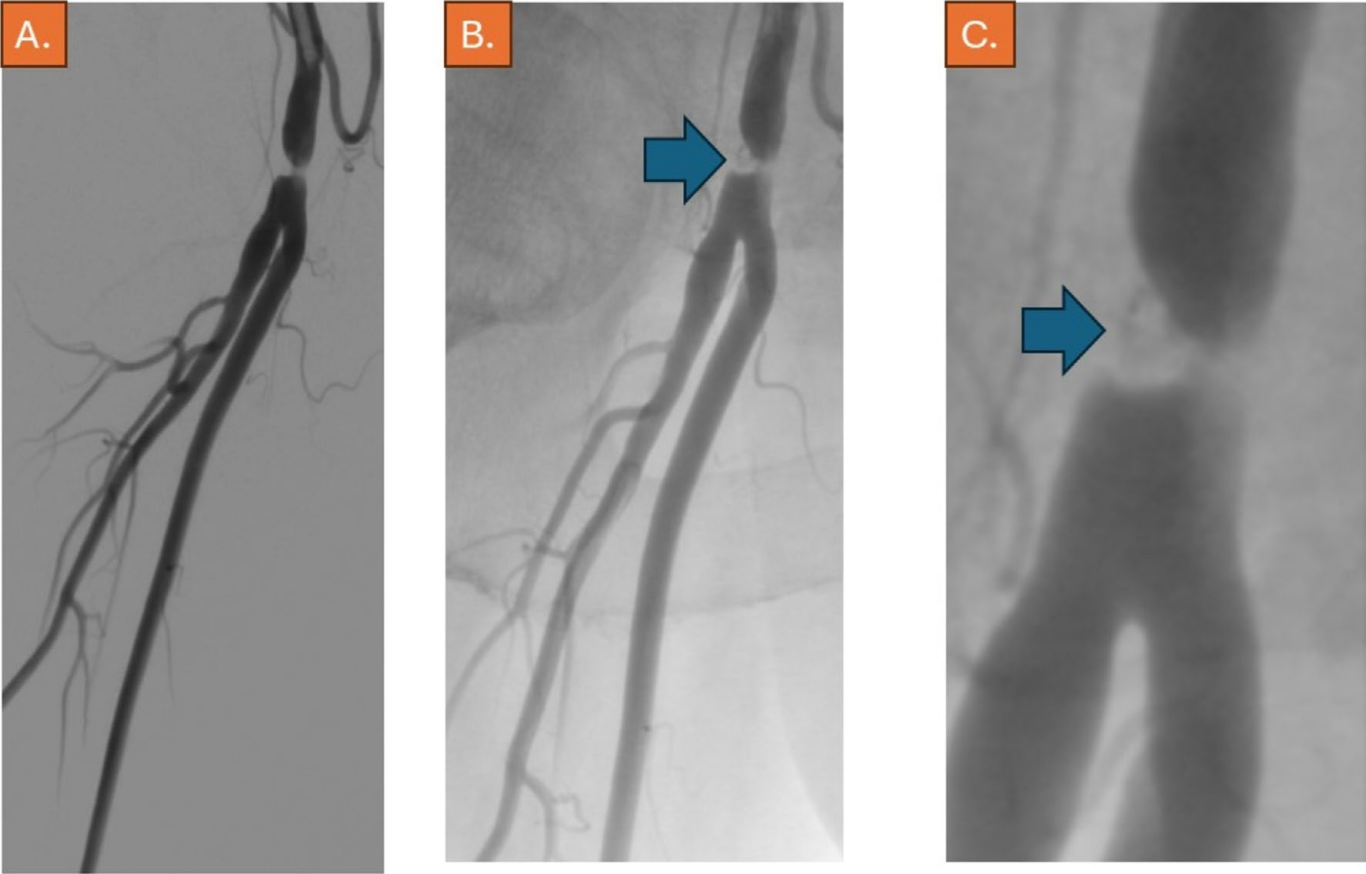
(A) Angiogram, 15° RAO angulation, showing the subtotal occlusion of the right common femoral artery, (B) same angulation with magnification, where the nitinol‐clip of the StarClose device can be depicted (blue arrow), (C) same angulation with higher magnification.

**FIGURE 3:**
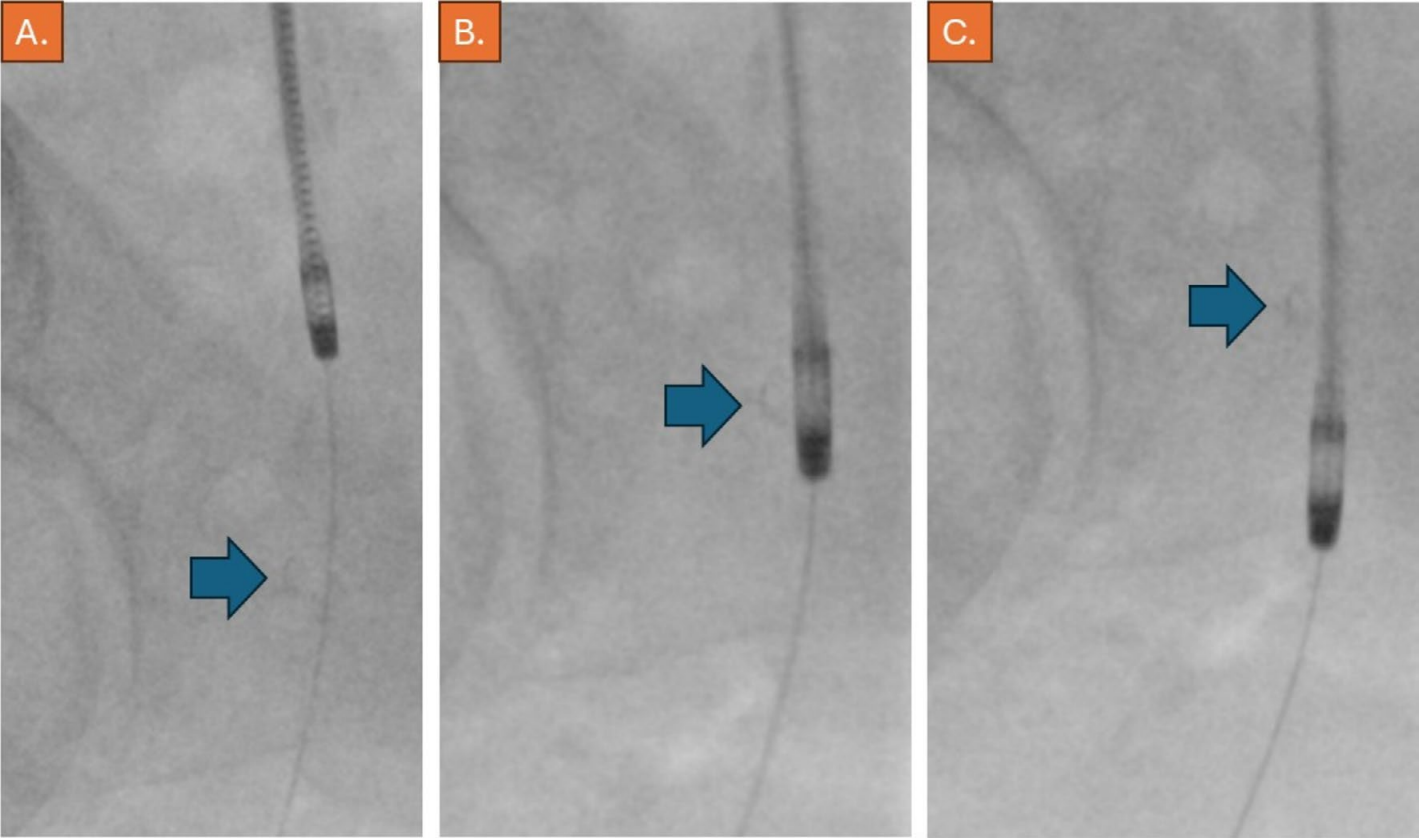
(A–C) Fluoroscopy, 15° RAO angulation, showcasing different passages of the subtotal occlusion at the right common femoral artery with the Rotarex 8F device (blue arrow pointing at the nitinol‐clip of the StarClose device).

Angiography was performed after the puncture of the contralateral CFA and cross‐over maneuver. Indeed, subtotal occlusion of the distal CFA was confirmed (Video S1), whereas image magnification confirmed the protrusion of the StarClose nickel‐titanium clip and possibly of parts of the vessel wall into the lumen of the artery, causing the occlusion (Figure 2A–C). Since the patient preferred immediate endovascular management, treatment was proceeded with using an 8F Rotarex thrombectomy device (BD Vascular, Straub Medical, Wangs, Switzerland), which was slowly and carefully advanced through the subtotal occlusion across the nickel‐titanium clip of the StarClose device, which was well visible during fluoroscopy (Figure 3A–C, Video S2). We anticipated that improper deployment within the vessel wall, followed by manual compression, likely resulted in intimal injury and subsequent thrombus formation, leading to subtotal occlusion of the artery.

**FIGURE 4:**
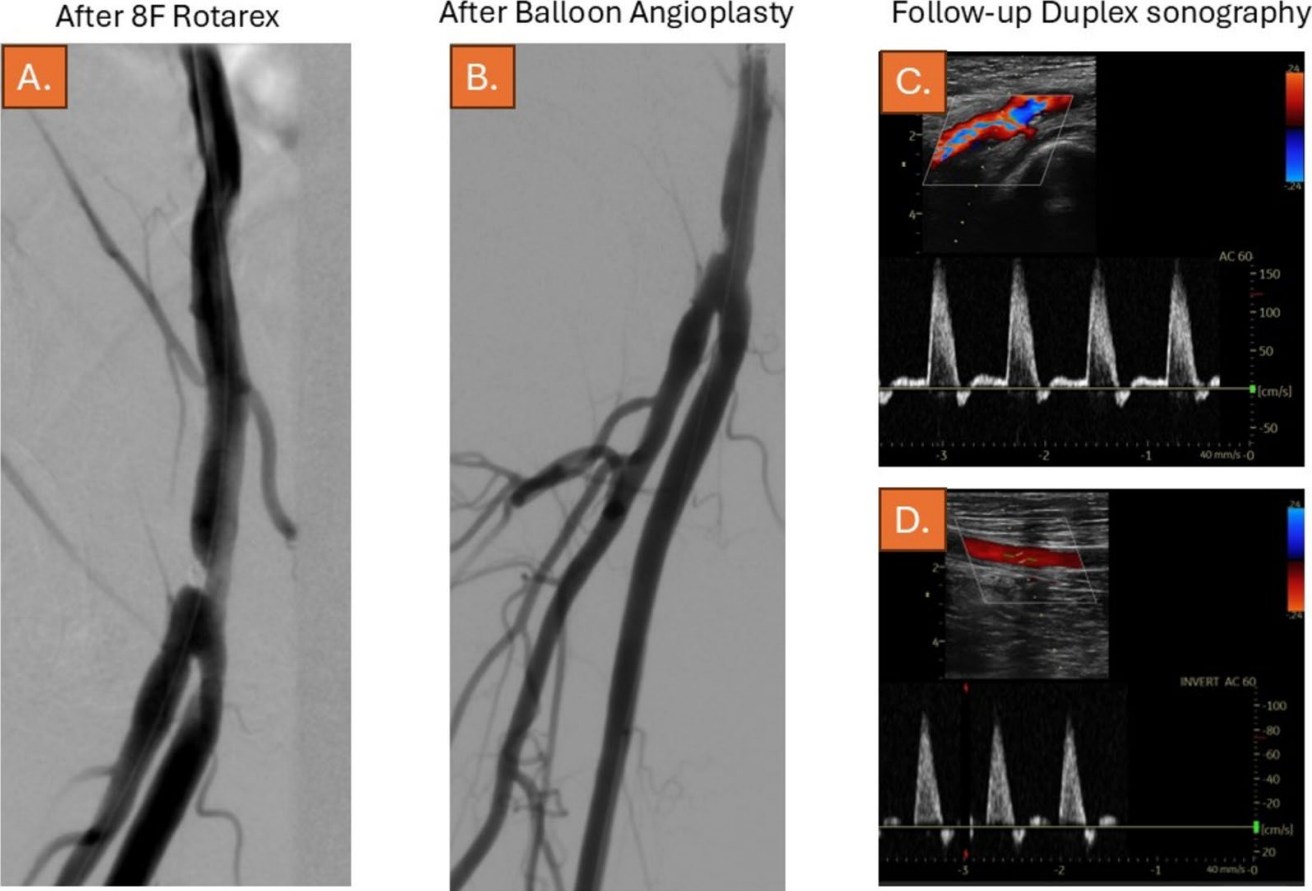
(A) Angiogram, 15° RAO angulation of the right common femoral artery, highlighting the luminal gain as an immediate result after use of the Rotarex device. (B) Angiogram, showing the result after additional DCB angioplasty of the vessel using a 7,0/40 mm Ranger DCB (Boston Scientific). Note the mild residual constriction at the adventitial site of the vessel wall caused by the nitinol clip. Duplex sonography after 4 weeks showed no residual stenosis of the CFA (C) and triphasic flow in the SFA (D).

After several passages, a lumen gain of 50% was noticed by angiography (Figure 4A, Video S3). Subsequently, drug‐coatedballoon (DCB‐) angioplasty (Ranger 6.0 × 60‐mm and 7.0 × 60‐mm balloons, Boston Scientific, Marlborough, MA, USA) was performed, resulting in further significant lumen gain and residual stenosis < 30%, obviating the need for additional stent placement (Figure 4B, Videos S4 and S5).

### 2.2 Outcome and Follow‐Up

The further course of the patient was stable, and she was discharged 2 days after the endovascular revascularization procedure. Dual platelet inhibition with aspirin and clopidogrel was continued for 5 months. Ischemic symptoms completely resolved, and duplex sonography after 4 weeks showed no residual stenosis of the CFA and triphasic flow in the SFA (Figure 4C,D).

## 3 Discussion

To our knowledge, this is the first case report on interventional treatment of a subtotal vessel occlusion caused by the StarClose vascular closure device. The StarClose device, designed to achieve rapid hemostasis after percutaneous interventions, particularly in the CFA, is generally regarded as safe and effective. However, complications such as arterial dissection, thrombosis, and occlusion, though infrequent, have been reported in rare cases, especially when the device is used in anatomically challenging or off‐label locations like in the SFA or the deep femoral artery [6, 7, 8].

The StarClose device uses a nitinol clip to approximate the arterial walls for hemostasis. While effective in the CFA, its application in smaller or calcified vessels like the SFA can lead to complications due to increased mechanical stress on thinner arterial walls. Studies have documented complications such as hematoma, pseudoaneurysm, and stenosis following StarClose use, with complication rates ranging from 0.7% to 7.4% depending on patient and procedural factors [7, 9]. Notably, for coronary interventions, the radial approach is strongly preferred for all patients since it is associated with significantly lower rates of bleeding and vascular complications [10]. In the present case, right radial access was attempted, but was not feasible due to strong vessel tortuosity, so the operator decided to switch to the right femoral access.

Previous reports have primarily described surgical interventions to address such complications. For instance, Gonsalves et al. reported a case of CFA laceration following StarClose deployment, requiring surgical repair [11]. Similarly, a case involving intimal injury and subsequent arterial occlusion made open surgical correction necessary [12]. On the other hand, our case demonstrates the feasibility of an endovascular approach, using a rotational athero‐thrombectomy device (Rotarex 8F) followed by DCB angioplasty, to manage a subtotal occlusion of the CFA caused by the inappropriately deployed StarClose device. The novelty of our case is associated with the use of debulking plus DCB and without stenting for solving this endovascular treatment‐related complication. The procedure was performed 3 weeks after the index procedure, due to an acute limb ischemia (ALI) occurring at the previous access site. Due to lumen gain after debulking and DCB angioplasty, no need for a stent implantation was necessary and a normal blood flow could be achieved. Notably, the CFA is an access site and a bypass anastomosis zone where the placement of permanent metallic implants is not the treatment of the first choice [13]. For this reason and due to sufficient lumen gain after debulking and DCB angioplasty, we preferred to leave the CFA without a stent and rather perform close surveillance of the patient by Duplex sonography.

In the past decade, emerging technical developments with endovascular therapy, such as atherectomy and thrombectomy, offer several advantages of minimally invasive treatment over surgical techniques. Different devices have been developed to achieve luminal gain without barotrauma, avoid dissections, and the need for stent implantation at the treated site. The Rotarex device provides simultaneous mechanical debulking and aspiration of the fragmented thrombotic material [14]. It has demonstrated high efficacy in ALI caused by thrombotic or embolic events, achieving revascularization rates higher than 90% when combined with adjunctive therapies. Its ability to minimize embolization risk makes it particularly suitable for treating occlusions caused by foreign‐body‐related thrombosis. Following thrombectomy, balloon angioplasty addresses residual stenosis caused by intimal dissection or vascular narrowing. In our case, and due to vessel injury, DCB was chosen for angioplasty to prevent re‐occlusion due to its additional anti‐restenotic action. In addition, we anticipated that debulking aided lumen gain and removal of soft plaque components and thrombus, which was also confirmed by the angiographic image after debulking and prior to balloon angioplasty.

Minimally invasive strategies offer potential benefits, including reduced recovery times and avoidance of the risks associated with open surgery. Development and refinement of complication management options, as described in our case report, are therefore useful, aiding further establishment of such endovascular techniques for the treatment of PAD. In addition, operator expertise and meticulous techniques during the deployment of vascular closure devices are crucial to minimize complication rates [15]. In addition, adherence to current studies and guidelines, which recommend the radial over the femoral access for coronary interventions [10], and encourage the use of ultrasonography‐guided puncture to reduce the risk of venous or failed punctures and vascular device‐associated complications, as in our case, is crucial [16, 17, 18]. Future studies should also address the long‐term outcomes of endovascular management for vascular closure devices‐related complications and help to develop standardized protocols for patient management in such cases.

In conclusion, our case demonstrates that endovascular treatment using Rotarex 8F thrombectomy followed by DCB angioplasty is an effective strategy for managing ALI caused by a vascular device‐induced CFA occlusion. This approach restores perfusion while minimizing procedural risks compared to traditional surgical methods. Further studies are needed to optimize treatment strategies and improve outcomes for patients experiencing such rare but significant complications from vascular closure devices.

## Author Contributions

**Theodoros Moysidis:** conceptualization, investigation, methodology, supervision, writing – original draft, writing – review and editing. **Daniel Herzenstiel:** conceptualization, formal analysis, investigation, methodology, validation, visualization, writing – original draft, writing – review and editing. **Grigorios Korosoglou:** conceptualization, formal analysis, investigation, methodology, resources, software, supervision, validation, visualization, writing – original draft, writing – review and editing.

## Consent

Herewith we declare that the patient gave her informed consent for the anonymous publication of all data acquired in our institution, including image material and all relevant data. Due to our administrative regulations with respect to data protection, we cannot send out or upload any documents where the ID, the signature, or the name of the patient can be identified. However, complete anonymization is provided with the article. All details have been removed from the case descriptions to ensure anonymity.

## Conflicts of Interest

The authors declare no conflicts of interest.

## Supporting information


**Data S1:** ccr370689‐sup‐0001‐DataS1.zip.

## Data Availability

The data that supports the findings of this study are available on reasonable request from the corresponding author. The data is not publicly available due to privacy or ethical restrictions.
