# Supplementary material for: Rotarex Debulking in a Subtotal Occlusion of the Common Femoral Artery After Intimal Protrusion Caused by a Vascular Closure (StarClose) Device
Source: Clin Case Rep. 2025 Aug 14;13(8):e70689. doi: 10.1002/ccr3.70689 (PMC12354975; doi:10.1002/ccr3.70689)
Supplement: Supplementary file 1 — Data S1: ccr370689‐sup‐0001‐DataS1.zip. [file CCR3-13-e70689-s001.zip › supinfo/transcriptional text for the videos.docx]

Video 1. Digital subtraction angiogram, showing subtotal occlusion of the CFA.

Video 2. Fluoroscopy images during Rotarex thrombectomy.

Video 3. Digital subtraction angiogram after several passages with the 8F Rotarex catheter.

Video 4. Digital subtraction angiogram after balloon angioplasty.

Video 5. Cine angiogram after balloon angioplasty, showing brisk flow to the foot of the patient.
